# Prevalence of orthorexia nervosa in university students and its relationship with psychopathological aspects of eating behaviour disorders

**DOI:** 10.1186/s12888-018-1943-0

**Published:** 2018-11-13

**Authors:** María-Laura Parra-Fernández, Teresa Rodríguez-Cano, María-Dolores Onieva-Zafra, María José Perez-Haro, Víctor Casero-Alonso, Elia Fernández-Martinez, Blanca Notario-Pacheco

**Affiliations:** 1 Faculty of Nursing , University of Castilla-La-Mancha, Ciudad Real, Spain; 2 Head of Mental Health, Castilla la Mancha Health Services, Ciudad Real, Spain; 3 Biostatech Advice, Training and Innovation in Biostatistics, S.L Santiago de Compostela, A Coruña, Spain; 4 School of Industrial Engineers, University of Castilla-La Mancha, Ciudad Real, Spain; 5 Faculty of Nursing, University of Castilla-La-Mancha, Cuenca, Spain

**Keywords:** Orthorexia nervosa, Eating disorders, University students, Psychological traits, Behavioral traits

## Abstract

**Introduction:**

Orthorexia nervosa (ON) is characterized by an obsession with healthy eating, which may lead to severe physical, psychological and social disorders. It is particularly important to research this problem in populations that do not receive clinical care in order to improve early detection and treatment.

**Objective:**

The aim of this study was to research the prevalence of ON in a population of Spanish university students and to analyze the possible associations between ON and psychological traits and behaviors that are common to ED.

**Method:**

A cross-sectional study with 454 students from the University of Castilla La Mancha, Spain. In total, 295 women and 159 men participated, aged between 18 and 41 years. The ORTO-11-ES questionnaire and the Eating Disorder Inventory (EDI-2) were used for this study. The chi squared test was used to compare the homogeneity among the different groups.

**Results:**

The scores on the ORTO-11-ES suggested that 17% of students were at risk of ON. The scores on the EDI-2 for the group at risk of ON were significant, compared to the remaining individuals, regarding their drive for thinness (17.1% vs 2.1%), bulimia (2.6% vs 0%), body dissatisfaction (26.3% vs. 12.4%), perfectionism (14.5% vs 4.8%), interoceptive awareness (13.2% vs 1.3%), asceticism (15.8% vs 3.7%) and impulsiveness (9.2% vs 1.9%).

**Discussion and conclusion:**

These findings suggest that many of the psychological and behavioral aspects of ED are shared by people who are at risk of ON. Future research should use longitudinal data, examining the temporal relationship among these variables or other underlying variables that may contribute to the concurrence of ED and ON.

## Introduction

The term ‘eating disorders’ (EDs) encompasses a variety of disorders characterized by abnormal eating behaviors associated with emotional difficulties. The EDs described in the fifth edition of the diagnostic and statistical manual of mental disorders (DSM-5) [1] may not be entirely applicable to specific populations due to the wide variability in the frequency, the time-period and the characteristics of each individual, limiting the application of available diagnostic criteria.

Orthorexia nervosa (ON) is described as an obsession for healthy food. This term was used for the first time by Bratman in 1997 [2]. People who suffer from this eating fixation undergo a monomania for healthy food without artificial additives and are more concerned with the quality of food than the quantity [3]. This extreme concern for food can lead to a disorder with many different levels of severity. These patients have important dietary restrictions, which are related to medical disorders that are potentially mortal associated with malnutrition, affective instability and social isolation [3].

To date, neither the diagnostic criteria published for ON [4, 5], nor the different studies available have given enough clarity to include this disorder in the DSM-5 [1], nor in the tenth edition of the International Classification of Diseases (ICD-10) [6]. Furthermore, some studies have related ON with obsessive compulsive disorders (OCD) [7–10]. Donini et al. performed a study, in which they developed and validated a questionnaire to detect the risk of suffering ON: the ORTO-15 [11]. The same study reported an association between ON and OCD. In addition, most of the literature consulted by the authors of this study, reveals clinical characteristics of ON that are common in EDs, in particular in anorexia nervosa (AN) [12–15]. A study by Brytek—Matera found that the participants who displayed a great level of concern with healthy foods also showed a positive correlation with satisfaction and/or the appearance of their body, and therefore this is one of the characteristics that is also found in patients with AN [16]. A study developed by Vandereycken et al. showed that ON is a disorder that is often referred and acknowledged by patients with ED. According to this study, 67% of professionals in charge of the treatment of these patients observed this phenomenon in their clinical practice, and 69% considered that the disorder warranted greater attention [17]. Both ED and ON are characterized by a lack of pleasure related with eating food and show a need for controlling the intake of food as a tool for improving their self-esteem and/or self-fulfillment, granting them a sense of control over their own life [18]. The difference between these two disorders is that, while people with orthorexia are focused on eating healthy and pure foods, preoccupied by quality, those who suffer from anorexia and/or bulimia are more concerned with the quantity of the foods they eat, rather than the quality of the same [19]. Vargas et al. point out that although the difference between both effectively resides in the final motivation, i.e. weight loss in AN or feeling healthy in the case of ON, similar social and psychological consequences may exist in both disorders [20]. Furthermore, some authors attempt to identify or clarify the existing relationship between some EDs and mental disorders [21]. Dell’Osso et al. propose the hypothesis that people at risk of suffering ON, besides sharing some traits with people who suffer autism spectrum disorders (ASDs) such as for example ritual-like behaviors when preparing food, may also share consequences such as the risk for social isolation [22].

Among the different studies available on the prevalence of ON, several questionnaires [11, 23, 24] have been used to determine the presence of the disorder. Most of these are based on the proposal by Donini et al., i.e. the ORTO-15 [11]. Depending on the instrument used and the populations in which the study is performed, the results of the prevalence rates vary. One of the first studies performed in Italy by Donini et al. in 2004 using the ORTO-15 demonstrated a prevalence of 6.9% in a population of 404 students [25]. Kinzl et al. used the original test by Bratman in a sample of 283 dieticians, and found that 34.9% of the population had a high risk of ON [10]. In a study involving 446 German university students conducted by Depa et al. employing the Düsseldorfer Orthorexie Skala (DOS) [23], a 3.3% estimated prevalence of ON was reported, together with a 9.0% prevalence for the risk of developing ON [26]. It is important to consider that most studies have been performed in non-clinical settings, and mainly on university students [8, 13, 19, 23, 26, 27].

Lifestyle habits and food consumption are developed since infancy and begin to establish themselves in adolescence and youth. The diet of youth, and especially that of university students is an important challenge, as it may involve important lifestyle changes [28]. The university population is an especially vulnerable group from the nutritional point of view, as they are beginning to take responsibility for their own dietary habits and they undergo a critical period in the consolidation of eating habits and behaviors [29]. Young adulthood (19–24 years) is an important developmental period for exploring and establishing our relationship with health habits, beliefs and eating norms, as well as for body image development [30]. Considering that many of the conditions and behaviors established during teenage years persist throughout life, adolescence and adulthood, these periods represent powerful developmental opportunities for evaluating predictors and risk factors for ED. These behaviors should be addressed due to their adverse consequences such as metabolic risks later on in adulthood. Improving our understanding of populations who do not receive clinical care such as people with a risk of ED is particularly important for early detection and treatment of ON [31, 32].

To date there is no data available on prevalence in the Spanish university population, or regarding the possible relations with characteristics that appear in other EDs.

Therefore, the aims of this study were to estimate the prevalence rate of ON in a Spanish university population with a tool that has been validated for this purpose and to determine the possible correlation of ON with psychological and behavioral aspects that appear in other EDs. The present study has considered indicators which are commonly associated with EDs: the body mass index (BMI) and sex, which will help us to clarify and further our understanding regarding this phenomenon.

## Method

### Study design and subjects

This cross-sectional study was planned and performed between January and May 2017, in Ciudad Real, Spain. We invited 800 university students from different faculties (Nursing, Law, Chemistry, Computer Science and Education), of which 454 university students participated (response rate: 56.75%) including 295 women and 159 men, aged between 18 and 51 years (mean age, 21.74 ± 4.73 years). The participants were recruited through informative talks delivered during university lectures in different faculties.

Data collection was performed via a questionnaire prepared by the researchers. The revised questionnaire was divided into three sections: (1) Sociodemographic characteristics; (2) the Eating Disorder Inventory-2 questionnaire (EDI-2) [33, 34]; and (3) the ORTO-11-ES [35, 36].

The University students voluntarily signed up to the study and they were asked to complete an online survey developed using the JotForm platform. It was assumed that the students who did not respond were within the same range of conditions as those who did. For ethical reasons, we were unable to research the causes which made these students decide not to participate.

### Ethical considerations

The participants did not receive any financial incentive to take part in the study. Participants were informed that their information was to be kept confidential and would only be used for scientific purposes, obtaining the written informed consent of participants. The ethical committee of the Castilla-La Mancha University Hospital approved the study (Number C-45), according to the ethical principles for medical research gathered in the Declaration of Helsinki [37].

### Measurements

#### Demographic information

The sociodemographic forms gathered information on the age, gender, height and weight of participants. The BMI of each participant was calculated based on the self-reported height and weight.

#### Eating disorder inventory (EDI-2)

This is a self-reported 91-item questionnaire, answered on a 6-point Likert-Type scale using a 3-point system where ‘sometimes’, ‘rarely’, and ‘never’, are assigned zeros while ‘often’, ‘usually’ and ‘always’ are assigned a score of 1, 2 and 3, respectively. The questionnaire is used to assess eating-disorder symptoms, attitudes and behaviors. It contains 11 subscales: drive for thinness, body satisfaction, bulimia, effectiveness, perfectionism, interpersonal disruption, interoceptive awareness, maturity fears, asceticism, impulse regulation and social insecurity. The sub-scale scores can be calculated by simply adding the scores of all the items of each specific sub-scale. The EDI-2 total score ranges from 91 to 546. We used a Spanish version of the scale validated by Corral, González, Pereña & Seis dedos (1998), which showed an internal consistency of 0.83–0.92 [34].

The EDI-2 is widely used in Spain and it has been demonstrated to be a valid instrument for the accurate diagnosis and detection of the risk of ED [38–40] in the Spanish population. We chose to use the EDI-2 based on its good psychometric properties, in both clinical settings and non-clinical samples [33] as well as the possibility it offers for separately assessing different dimensions [41].

#### ORTO-11- ES questionnaire

The ORTO-15 questionnaire was originally developed in Italian [11]. This tool consists of 15 self-report multiple-choice items using a 4-point Likert-type scale (always, often, sometimes, never) to measure three underlying factors related to eating behavior: cognitive-rational (items 1, 5, 6, 11, 12 and 14), clinical (items 3, 7, 8, 9 and 15) and emotional aspects (items 2, 4, 10 and 13). It is used to investigate obsessive behavior related to the selection, preparation, habits of food consumption and attitudes towards healthy food. The lower the score, the higher the indication of a behavior or attitude related to orthorexia. The Italian group [11] suggested a cut-off score of 40 points, whereby scores below this figure indicate ON related behavior.

For the present study, we have used the ORTO-11-ES [35] as a tool for assessing ON. This tool is based on a structure of three factors for the abbreviated 11-item version, and has demonstrated an appropriate internal consistency (Cronbach’s alpha = 0.80). Furthermore, the test has demonstrated a good predictive capacity for a threshold value of < 25 (79.5% effectiveness, 75% sensitivity and specificity 79.6%).

### Statistical analyses

An exploratory statistical analysis of all the demographic variables and the ON-tendencies was carried out. Quantitative features were described by the median and the inter-quartile range (IQR) and qualitative variables were described using frequencies and percentages.

To identify the score differences among the different groups (individuals with ON tendencies and individuals without ON tendencies) and without an assumption of normality for scores and small sample sizes (*N* < 30) for some of the subgroups, the Wilcoxon-Mann-Whitney (W-M-N) and Kruskal-Wallis (K-W) tests for independent samples were performed.

For each feature (gender, smoker and BMI), the prevalence of ON was calculated as the proportion of individuals of a certain population that are under risk of suffering ON in this period.

This analysis has also been performed for each sub-scale of the Eating Disorder Inventory-2, i.e. for Drive for Thinness, Bulimia, Body Dissatisfaction, Ineffectiveness, Perfectionism, Interpersonal Distrust, Interoceptive Awareness, Maturity Fears, Asceticism, Impulse Regulation and Social Insecurity. Moreover, a correlation analysis was performed between the scores of the sub-scales of the EDI-2 and the scores of the ORTO-11-ES, using the Spearman coefficient.

The significance level was established at *p* < .05 for all cases. The R statistical software was used to perform all the statistical analyses [42].

## Results

The sample included 454 students recruited from the Castilla-La Mancha University, and who voluntarily answered the questionnaire. A summary of the demographic variables is shown in Table 1.

**Table 1:** Descriptive analysis of the sample

Qualitative variable		Frequency
Smoker	Yes	92 (20.30%)
	No	362 (79.70%)
Sex	Female	295 (65.00%)
	Male	159 (35.00%)
Marital Status	Single	444 (97.8%)
	Married	10 (2.20%)
Quantitative variable		Median (IQR)
Age		20.00 (19.00–22.00)
Body Mass Index		22.21 (20.31–24.50)

The mean score obtained by the total participants regarding the ORTO-11-ES questionnaire was 27.78 and the standard deviation was ±3.34. The cut-off score was established at < 25 [35] ranging from 16 to 36 points, with 76 (17%) participants under risk of suffering ON.

The location parameter for the age, in those who were under a true risk of suffering ON, was not significantly different from those who were not under a real risk (W-M-*N* = 12,917, *p* = .16), neither was it significant for gender (W-M-*N* = 22,916, *p* = .69). The BMI variable was categorized into three groups, 1) below 18.5 (thinness); 2) 18.5, 24.9 (normal weight); 3) 25–41 (obesity). The differences of the ORTO-11-ES scores among the three groups were also non-significant (K-W χ2(2) = 1.9466 *p* = .38). On the other hand, statistical differences were found for smokers (W = 13,462, *p* = .00).

### Prevalence and features of orthorexia nervosa

The prevalence of ON is significantly higher in women, as reported in the Italian population. [43]. There are no significant differences among the other groups. (See Table 2).

**Table 2:** Prevalence of Orthorexia for each feature

Feature	Prevalence of ON (%)	χ^2^	DF	*p*-value
Male	11.9	4.03	1	**.04**
Female	19.3			
Smoker	18.0	1.89	1	.17
Non-smoker	12.0			
BMI: Thinness	25.0	1.95	2	.38
BMI: Normal weight	16.2			
BMI: Obesity	15.4			

Bold data indicates statistically significance (*p* < .05) indicated bold data

Concerning the ED, the analysis suggests that the individuals at risk of suffering ON have a higher prevalence rate of drive for thinness (17.1% vs 2.1%, χ2(1) = 32.22, *p* = .00), bulimia (2.6% vs 0%, χ2(1) = 9.99, p = .00), body dissatisfaction (26.3% vs. 12.4%, χ2(1) = 9.6, p = .00), perfectionism (14.5% vs 4.8%, χ2(1) = 9.98, p = .00), interoceptive awareness (13.2% vs 1.3%, χ2(1) = 27.74, p = .00), asceticism (15.8% vs 3.7%, χ2(1) = 17.12, p = .00) and impulse regulation (9.2% vs 1.9%, χ2(1) = 11.46, p = .00) than people who are not at a risk of suffering this disorder (see Table 3).

**Table 3:** Prevalence of eating disorders in a population at risk of ON and in a healthy population

Dimension EDI-2	Orthorexia Nervosa		χ^2^	df	*p*-value
	Yes (%)	No (%)			
Drive for thinness	17.1	2.1	32.22	1	**.00**
Bulimia	2.6	0.0	9.99	1	**.00**
Body Dissatisfaction	26.3	12.4	9.69	1	**.00**
Ineffectiveness	9.2	4.0	3.77	1	.05
Perfectionism	14.5	4.8	9.98	1	**.00**
Interpersonal Distrust	6.6	8.7	0.38	1	.54
Interoceptive Awareness	13.2	1.3	27.74	1	**.00**
Maturity Fears	22.4	14.3	3.13	1	.08
Asceticism	15.8	3.7	17.12	1	**.00**
Impulse regulation	9.2	1.9	11.46	1	**.00**
Social Insecurity	11.8	8.5	0.88	1	.35

Bold data indicates statistically significance (*p* < .05) indicated bold data

In addition, a correlation analysis of the ED sub-scale scores and the ON scores has been carried out (see Table 4). Due to the lack of normality in all the scores, the Spearman correlation coefficient was calculated. All of these tests were negative and statistically significant (*p* < 0.05). The negative sign indicates that, in general, high values of the ED subscales correspond to low values for the ON scores. The highest (negative) correlation coefficient (− 0.564, *p* = 0.00) was found between drive for thinness and the ON score.

**Table 4:** Correlation analysis of the EDI-2 sub-scales scores and the ON scores

Dimension EDI-2	Spearman coefficient	*p*-value
Drive for thinness	−0.564	0.00
Bulimia	−0.260	0.00
Body Dissatisfaction	−0.347	0.00
Ineffectiveness	−0.228	0.00
Perfectionism	−0.248	0.00
Interpersonal Distrust	−0.147	0.00
Interoceptive Awareness	−0.344	0.00
Maturity Fears	−0.113	0.02
Asceticism	−0.168	0.00
Impulse regulation	−0.210	0.00
Social Insecurity	−0.148	0.00

## Discussion and conclusion

The aim of the present study was to determine the prevalence of suffering ON and its possible relation with psychological and behavioral aspects of ED in a population of Spanish university students. We used the ORTO-11-ES [35], our findings reveal that 17% (76 students) of the sample presented a high risk of suffering from ON. This percentage is far from that obtained in the unique study on ON conducted on a sample of the Spanish population, where the results showed a prevalence of 86% [44]. However, this pilot study did not use a validated translation of the original ORTO-15 [11], rather it used the English version on a sample of 136 ex-students of Ashtanga yoga. Moreover, the age range of participants in the aforementioned study was higher than the age of university students [44]. Dunn et al. [45] found that 1.0% of students in the United States suffered from ON and suggested that 10.0% of the population was at risk of developing this disorder. In Italian populations, different studies place the prevalence of ON in a range of between 6.9 to 57.6% [25, 46]. In Turkey, a validated adaptation of this tool, the ORTO-11, showed a prevalence of approximately 45% in different studies with samples of university healthcare students [8, 13]. The greatest prevalence, 74.2%, was reported in a study conducted in Hungary, also using a translated and validated version of ORTO-11-Hu in a sample of university students [19]. Considering the varying results obtained across different countries, in part, some of these differences may be explained by socio-cultural factors, being closely related with the eating habits linked to the culture of each country [7, 47]. However, other authors attribute these differences to the structure of the questionnaire itself rather than cultural problems [48]. Furthermore, when interpreting these results, it is important to consider that the prevalence is linked to the interpretation of different versions of a self-reported questionnaire, which have used different cut-off points [11, 36, 49, 50] .

A significant correlation between ON and the psychopathological characteristics of other EDs, was observed based on the variables included in the EDI-2 subscales: drive for thinness, bulimia symptoms, body dissatisfaction, perfectionism, interoceptive awareness, asceticism and impulsiveness. These findings highlight the possible relation between the risk of suffering ON and the diagnosis of ED. Some of our results reinforce findings from previous studies [51, 52]. In a sample of 220 university students, Barnes et al. [51] concluded that there was a positive relation between ON and other ED, regarding the body image attitude and the perfectionist personality of these individuals. Also, having a personal history of having suffered an ED was found to be a strong predictor for ON. Another study, also along these lines, performed with 459 university students in the United States, showed a positive correlation between ON and perfectionism [52]. Two further clinical studies also highlighted the close relation between ED and ON [23, 53]. One of these, conducted in Germany with a sample of 1122 hospitalized patients with psychiatric diagnoses found positive correlations between ON and the dimensions drive for thinness, interoceptive awareness and asceticism in patients diagnosed with ED [53]. The second study was performed with another tool for the detection of ON: the Dußßdorfer Orthorexie “DOS” scale [23]. This study included a sample of 1340 participants and found positive correlations with the EDI-2 subscales of thinness, bulimia and body dissatisfaction, suggesting proximity between ON and ED [23]. Currently, there is much debate surrounding the relationship between AN and ON, ranging from how to classify and differentiate these disorders, in some cases considering ON as a new disorder, or a subset of AN [53]. It is well known that undertaking weight-loss diets can lead certain individuals towards adopting extreme eating habits. There is a large coincidence between supposedly ‘healthy’ foods and generally ‘slimming’ foods which can lead individuals towards a confusion that is difficult to manage [23]. At times, this may lead to an obsession with healthy eating, until individuals adopt a more severe pathology, such as AN [17]. On the other hand, the opposite hypothesis can lead us to affirm that an orthorexic behavior can be interpreted as a phase or a tendency in patients who have been previously diagnosed with ED and are in a recovery phase, and who, displaying an improvement of symptoms, can end up developing orthorexic behaviors [18, 41]. These findings emphasize how concerns regarding healthy eating can act as a predisposing factor for developing AN or Bulimia nervosa (BN), and as a key residual symptom which may potentially favor relapses of the illness [54, 55]. Only with further research studies on clinical samples can we reveal the relationship between these two pathologies, and determine whether ON may be a factor that predicts the development of AN or viceversa.

Another aim of our study was to explore the relationship of ON with variables such as gender, age, weight, and body mass index. We found significant differences for the mean score on the ORTO-11-ES [35] scale in the female population. If we compare this with other studies, this result is striking as in most studies no differences were found regarding gender [8, 44, 52, 56]. In the study by Donini et al., they concluded that men are more sensitive to suffering from this problem [11]. This result has been repeated in one other study performed on a sample of Turkish students [13, 25]. However, there are other studies, which, like ours, report a greater proportion of women at risk of developing ON [7, 13, 57]. Although the gender difference of ON is harder to detect, in part, due to the lack of research in clinically diagnosed individuals [58], undoubtedly, gender is a critical factor in many aspects of life, including the attitudes and perceptions of one’s body image [59]. Indeed, there are a series of characteristics related to the internalization and externalization of emotions which may explain the different prevalence rates by gender in many mental illnesses [60].

Regarding the BMI, our results failed to find a significant correlation of the same with ON, a finding that supports most previous studies performed in different populations [56, 61]. In a study conducted by Aksoydan et al. in a population of 94 Turkish artists, no differences were found between the mean ORTO-15 score and the BMI [56]. Also, another study performed in Poland with 400 participants aged between 18 and 35 years failed to find a significant correlation with the BMI [61]. Varga et al. found that the association between the ON scores and the BMI was statistically significant, albeit insignificant [19]. Some authors suggest that the BMI can predict orthorexic behaviors in combination with other variables such as medical reasons, diet and healthy nutrition [7]. In contrast, another study also performed in Turkey on 878 medical students with a mean age of 21.3 ± 2.1 years found that, as the BMI increased, the ON score decreased, and, therefore, the risk of orthorexia nervosa increased [27]. Some authors justify this on the basis that overweight and obesity can expose the individual to humiliation and force the person to diet and consume healthy foods [13] .

Although this study is one of the first to examine the prevalence of ON in Spain, there are several limitations worth considering. First, the results do not provide information on the mechanisms that underlie the relationship between ON and EBD; for example, by considering other underlying factors such as biological factors, and personality, which could contribute to the high concurrence of these behaviors. Due to the cross-sectional design of this study, we cannot determine the time course of the development of EDs and ON. Therefore, by considering ON as a potential risk factor for developing an ED, a more complete longitudinal study is necessary in the future. Despite these limitations, the current study focuses on a gap in the literature regarding ON and EBD, broadly demonstrating the relationship between these.

Our results highlight the long path ahead for the scientific community, in order to recognize that ON can be included as another diagnosis within eating disorders. Additional studies are needed to describe the behavior of people with orthorexia (i.e. their etiology, diagnosis, treatment and the prevention of the same). On the other hand, studies on these subjects provide the health professional with the information necessary to be able to identify individuals with orthorexic behavior and thus provide appropriate treatment to derive the patient towards the most appropriate resource.

## Abbreviations

AN: Anorexia nervosa
BMI: Body mass index
BN: Bulimia nervosa
DOS: Düsseldorf Orthorexie Skala
DSM-5: Diagnostic and statistical manual of mental disorders
ED: Eating disorder
EDI-2: Eating Disorder Inventory
ICD-10: International Classification of Diseases
IQR: Inter-quartile range
OCD: Obsessive compulsive disorders
ON: Orthorexia nervosa
ORTO-11-ES: Spanish version Test for the diagnosis of Orthorexia nervosa
ORTO-15: Test for the diagnosis of orthorexia
